# A Near-Optimal Distributed QoS Constrained Routing Algorithm for Multichannel Wireless Sensor Networks

**DOI:** 10.3390/s131216424

**Published:** 2013-12-02

**Authors:** Frank Yeong-Sung Lin, Chiu-Han Hsiao, Hong-Hsu Yen, Yu-Jen Hsieh

**Affiliations:** 1 Department of Information Management, National Taiwan University, No. 1 Section 4, Roosevelt Road, Taipei City 106, Taiwan; E-Mail: yslin@im.ntu.edu.tw; 2 Department of Information Management, Shih Hsin University, No. 1, Lane 17, Mu-Cha Road, Section 1, Taipei City 116, Taiwan; E-Mails: hhyen@cc.shu.edu.tw (H.-H.Y.); d98725001@ntu.edu.tw (Y.-J.H.)

**Keywords:** Wireless Sensor Networks, Wireless Visual Sensor Networks, system perspective, user perspective, QoS, Near-Optimal Distributed QoS Constrained (NODQC), routing algorithm, Lagrangian Relaxation

## Abstract

One of the important applications in Wireless Sensor Networks (WSNs) is video surveillance that includes the tasks of video data processing and transmission. Processing and transmission of image and video data in WSNs has attracted a lot of attention in recent years. This is known as Wireless Visual Sensor Networks (WVSNs). WVSNs are distributed intelligent systems for collecting image or video data with unique performance, complexity, and quality of service challenges. WVSNs consist of a large number of battery-powered and resource constrained camera nodes. End-to-end delay is a very important Quality of Service (QoS) metric for video surveillance application in WVSNs. How to meet the stringent delay QoS in resource constrained WVSNs is a challenging issue that requires novel distributed and collaborative routing strategies. This paper proposes a Near-Optimal Distributed QoS Constrained (NODQC) routing algorithm to achieve an end-to-end route with lower delay and higher throughput. A Lagrangian Relaxation (LR)-based routing metric that considers the “system perspective” and “user perspective” is proposed to determine the near-optimal routing paths that satisfy end-to-end delay constraints with high system throughput. The empirical results show that the NODQC routing algorithm outperforms others in terms of higher system throughput with lower average end-to-end delay and delay jitter. In this paper, for the first time, the algorithm shows how to meet the delay QoS and at the same time how to achieve higher system throughput in stringently resource constrained WVSNs.

## 1. Introduction

**Figure 1.**
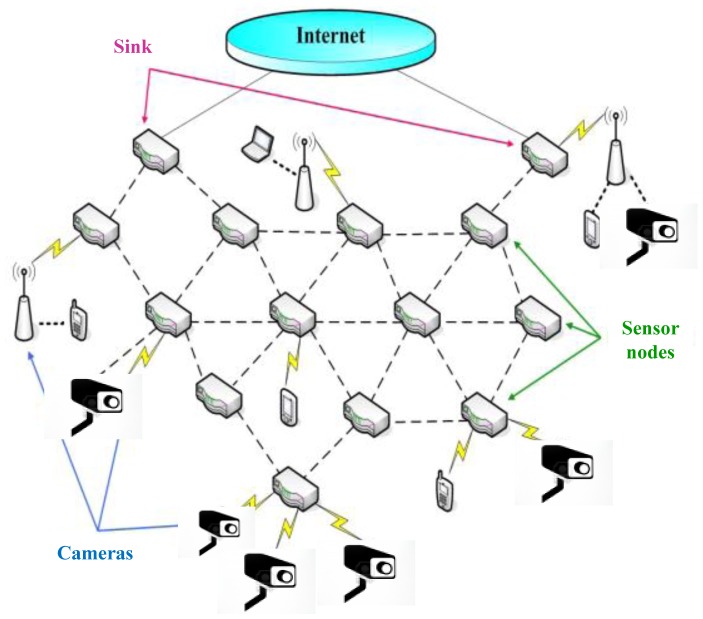
Wireless Visual Sensor Networks.

In recent years, Wireless Visual Sensor Networks (WVSNs) have emerged as an interesting field. Popular applications are environmental monitoring, seismic detection, military surveillance, medical monitoring, video surveillance, or the Internet of Things (IoT), *etc.* [1]. IoT integrates a part of the Future Internet and extends network capabilities to Machine-to-Machine (M2M) communications by a dynamic network infrastructure with self-organization techniques. As compared to a WSN, the camera equipped sensor nodes in WVSNs can capture, process and transmit the real time visual data (images, video). Since the visual data is much larger and complicated than scalar data, delay Quality of Service (QoS) is a challenging issue resource for constrained WVSNs. WVSNs attract more attention on how the camera sensor nodes can cooperatively pass their data more efficiently with minimum delay through the network on a large scale or under realistic physical environmental conditions [2,3]. The architecture of WVSNs is shown in Figure 1.

In WVSNs for video surveillance, the delay is a very important QoS metric, and the delay is highly dependent on the routing strategies. However, delay routing in WVSN is more difficult than in WSN due to the fact visual data is much larger and complicated than scalar data. Furthermore, battery-limited powered and resource (channel-, CPU-) constrained camera nodes make this delay routing problem more challenging in WVSNs. [4]. Basically, for real-time video surveillance applications, meeting the end-to-end delay QoS is the most important criteria. However, due to the limited resources of WVSNs, delay QoS routing decisions should also consider overall resource utilization so that the future video surveillance applications could be also satisfied. Hence, besides meeting the end-to-end delay QoS for each user, minimizing the average delay in WVSNs is also another crucial performance metric. In this paper, for the first time, we consider the delay QoS routing strategy in WVSNs to meet the end-to-end delay QoS for each application and at the same time minimize the average delay.

## 2. Literature Survey

Delay QoS routing is a challenging issue in WVSNs. Traditional approaches for wireless sensor networks are not efficient or even feasible in a WVSN due to three reasons: first of all, visual data are much bigger and complicated than scalar data, and processing and transmitting visual data will consume much more system resources than scalar data. Secondly, WVSNs need efficient collaborative image processing and coding techniques that can exploit correlation in data collected by adjacent camera sensor nodes. Video coding/compression that has low complexity, produces a low output bandwidth, tolerates losses, and consumes as little power as possible is required. Third, it needs to reliably send the relevant visual data from the camera sensor nodes to aggregation nodes or the sink node in an energy-efficient way [1,2]. This issue is related to the transmission technique that consists of the routing assignment to provide energy efficient and stable routes that meet the end-to-end QoS guarantees [2–4].

In wireless visual sensor networks for real time video surveillance, sensor nodes need to capture and forward the packets to the sinks within an acceptable delay under the limited resource constraints, including embedded vision processing, data communication, and battery energy issues. It is a challenging issue since it needs to meet the end-to-end delay for each application and at the same time optimize the system resource utilization by minimizing the average system delay so that more real-time applications can be granted access in the near future. Existing research on wireless sensor networks address the link scheduling techniques [5], channel assignments [6–8], routing algorithms [9,10], or resource allocations [11–13]. Some of the well-known routing protocols, such as *Ad hoc* On-demand Distance Vector (AODV), Dynamic Source Routing (DSR), Optimized Link State Routing (OLSR), and Destination Sequenced Distance Vector (DSDV) are the protocols applied to *Ad hoc* networks, such as MANETs, WMNs, VANETS, *etc.*, that consist of hosts interconnected by routers without a fixed infrastructure that can be arranged dynamically [14]. Even though like in the MANETs, there is no centralized AP or base station in WVSN, sensor nodes are also the relay nodes to forward the data to the sink node. However, without addressing the delay QoS, these approaches are not applicable to real-time applications. In the following, we survey existing approaches on shortest path routing, QoS routing, and routing algorithms. To the best of our knowledge, there is no research addressing the end-to-end delay from the user perspective and at the same time optimizing the system resource utilization by minimizing the average system delay.

### 2.1. Shortest Path Routing

The routing algorithm is determined by the shortest path and the routing decision for the O-D pair. When a new route is established and all traffic still follows the pre-existing path, this phenomenon is called “session routing” because a path remains in force for the entire user session. The routing metric (*i.e.*, cost) is used to define the path length and can be measured in terms of hops, the mean delay, and even the extent of flow deviation. The mean delay can be used to find the fastest path for routing, and flow deviation can be applied to system optimization routing where each route has equal flow deviation.

### 2.2. QoS Routing

Quality-of-Service (QoS) is a major issue that can be divided into several factors including the reliability, mean delay, delay jitter, and bandwidth of packet transmissions [15]. Controlling QoS is a challenge faced by the routing of multihop wireless sensor networks with interference [16]. In multichannel wireless sensor networks, transmitted packets may collide in an interference environment if the nodes and neighbors within transmission range use the same channel. This adversely affects system performance and the QoS. Different types of applications have different QoS standards. As such, the QoS routing problems differ by the QoS types requirements of end users and therefore require careful control of channel assignment within an interference environment. By using different and non-overlapping channels, each node can freely communicate with its neighboring nodes (*i.e.*, the nodes within transmission range) without interference. As noted in [15], using multiple channels instead of a single channel in multihop wireless sensor networks has been shown to not only reduce packet contention probability in non-overlapping channels, but to also dramatically improve the network throughput. A channel assignment strategy is necessary to efficiently use multiple channels with commodity hardware; besides, the channel assignment can be changed with significant changes in traffic load or network topology [7,8].

### 2.3. Routing Protocols

Routing protocols are categorized into table driven and on-demand, based on route calculation. Researches indicate that a Mobile *Ad hoc* NETwork (MANET) has several characteristics: (1) dynamic topologies; (2) bandwidth-constrained links; (3) energy constrained operation; and (4) limited physical security. Therefore the routing protocols for wired networks cannot be directly used for wireless networks [14,17,18]. The characteristics (1), (2) and (3) are similar to WVSNs, which is the driving force to design a protocol to apply to this case.

Proactive (table-driven) protocols are based on periodic exchange of control messages and maintaining routing tables. These protocols maintain complete information about the network topology locally. On the other hand, reactive (on-demand) protocols try to discover a route only on-demand, when it is necessary. These protocols usually take more time to find a route compared to proactive protocols. The following sections briefly introduce three well known protocols applied to MANET: Optimized Link State Routing (OLSR), *Ad hoc* On-demand Distance Vector (AODV), and Destination Sequenced Distance Vector (DSDV). The delay, throughput, control overhead and packet delivery ratio are the four common measures used for the comparison of the performance of the above protocols [18].

#### 2.3.1. Optimized Link State Routing (OLSR) (Table-Driven)

OLSR is an optimization of a pure link state algorithm for mobile *Ad hoc* networks that used the concept of Multi-point Relays (MPR) for forwarding control traffic. It is a proactive link-state routing protocol, which uses hello and topology control (TC) messages to discover and then disseminate link state information. OLSR is intended for diffusion into the entire network. The MPR set is selected such that it covers all nodes that are two hops away. The forwarding path for TC messages is not shared among all nodes, but vary depending on the source, and only a subset of nodes source link state information, and not all links of a node are advertised but only those that represent MPR selections.

Being a proactive protocol, routes to all destinations within the network are known and maintained before use. Having the routes available within the standard routing table can be useful for some systems and network applications as there is no route discovery delay associated with finding a new route [19].

#### 2.3.2. *Ad hoc* On-demand Distance Vector (AODV) (On-Demand)

AODV is a combination of on-demand and distance vector hop-to-hop routing methodology [7]. A connection broadcasts a request when a node needs to know a route to a specific destination. The route request is forwarded by nodes, and records a reverse route for itself to the destination. When the request reaches a node with a route to the destination it creates a backwards message which contains the number of hops that are required to reach the destination.

All nodes that participate in forwarding this reply to the source node create a forward route to the destination. This route created from each node from source to destination as a hop-by-hop state and not the entire route as in source routing [18].

The main advantage of this protocol is having routes established on demand and that destination sequence numbers are applied to find the latest route to the destination. The connection setup delay is lower. One disadvantage of this protocol is that intermediate nodes can lead to inconsistent routes if the source sequence number is very old and the intermediate nodes have a higher but not the latest destination sequence number, thereby having stale entries. Also, multiple route reply packets in response to a single route request packet can lead to heavy control overhead. Another disadvantage of AODV is unnecessary bandwidth consumption due to periodic beaconing [18].

#### 2.3.3. Destination Sequenced Distance Vector (DSDV) (Table-Driven)

DSDV is a hop-by-hop distance vector routing protocol. It is a table-driven routing scheme for *Ad hoc* mobile networks based on the Bellman-Ford algorithm. A routing table listing the “next hop” is maintained in each node for the reachable destination. Number of hops to reach destination and the sequence number are assigned by destination node. Each entry in the routing table contains a sequence number used to distinguish older routes from newer ones and thus avoid loop formation. The nodes periodically transmit their routing tables to their immediate neighbors. Routing information is distributed between nodes by sending full dumps infrequently and smaller incremental updates more frequently, so the updating process is both time- and event-driven. DSDV requires a regular update of its routing tables, which uses up battery power and a small amount of bandwidth even when the network is idle. Whenever the topology of the network changes, a new sequence number is necessary before the network re-converges. DSDV is not suitable for highly dynamic networks [20]. Table 1 summarizes a comparison of these routing protocols.

### 2.4. Motivation and Paper Organization

For real-time WVSN applications, the routing strategies should meet the end-to-end delay requirements and at the same time optimize the system resources so that more real-time applications could be admitted in the near future. To the best of our knowledge, there is no existing literature addressing these two issues at the same time. In this paper, for the first time, we propose WSN routing strategies that meet the end-to-end delay requirement and ate the same time optimize the system resources by minimizing the average delay. The rest of this paper is organized as follows: in Section 3, we propose the routing mathematical model to satisfy the end-to-end delay and minimize the average delay in the WSN. In Section 4, we present the solution approaches for our model and develop a heuristic to get a primal feasible solution. In Section 5, computational experiments are performed to verify the solution quality of our approach. Finally, we conclude this paper and outline future research in Sections 6 and 7.

## 3. Problem Formulation

### 3.1. Mathematical Modeling

The environment considered here is multichannel WSNs. This paper addresses the problem of determining a good QoS with the average cross-network packet delay while taking “user perspective” and “system perspective” into consideration. The following are details of the problem identification:

**Assumptions:**1.The channel assignment for each sensor node is fixed for a long period.2.Each sensor node is stationary.3.Each sensor node is equipped with multiple wireless interfaces, each of which operates on an individual and non-overlapping channel.4.Each sensor node can simultaneously communicate with its neighbors within transmission range without interference from the use of different channels for each link.5.A virtual node is added as the destination node to only connect to the sink via wired-line.6.All flows are transmitted to this virtual node via the sink.**Given:**1.The set of links.2.The set of sensor nodes.3.The link capacity of each link.4.The number of interference links of each link.5.The traffic requirement for each O-D pair.**Objective:**To minimize the average cross-network packet delay of the WSNs.**Subject to:**1.QoS constraints.2.Path constraints.3.Capacity constraints.4.Flow constraints.**To determine:**The minimum the average cross-network packet delay of the WSNs take system perspective and user perspective into consideration for each O-D pair.


We propose a channel assignment heuristic and routing algorithm to maximize the transmission rate for each node and enhance system performance by WVSN planning. A similar formulation that aimed to minimize the average cross-network packet delay subject to end-to-end delay constraints for users was proposed by Yen and Lin [21]. This paper's formulation can be extended to use queue models. Once the channel is assigned to the link, two nodes will use the assigned channel to communicate with each other until the channel assignment is changed (*i.e.*, static channel assignment). Every link is assumed to be fairly used at the scheduling phase, meaning the average time data transmission over each link is equal. In multichannel WVSNs, link capacity degrades because other links use the same channel in the interference range. Therefore, we divide the link capacity by the number of interference links in the following formulation (*i.e.*, $c_{l}=\frac{c_{l}}{I(l)}$) [6].

The following notations list the given parameters and the decision variables of our formulation, as illustrated in Tables 2 and 3.



**Objective Function (IP):**
$\min\underset{l\in L}{\text{∑}}D_{l}(g_{l})g_{l}$(IP)**Subject to:**
$\underset{l\in L}{\text{∑}}D_{l}(g_{l})y_{wl}\leq D_{w}$∀*w* ∈ *W*(IP 1)
$\underset{p\in P_{w}}{\text{∑}}x_{p}=1$∀*w* ∈ *W*(IP 2)
$\underset{p\in P_{w}}{\text{∑}}x_{p}\delta_{pl}\leq y_{wl}$∀*w* ∈ *W*, ∀*l* ∈ *L*(IP 3)
$0\leq g_{l}\leq \frac{C_{l}}{I(l)}$∀*l* ∈ *L*(IP 4)
$\underset{p\in P_{w}}{\text{∑}}\underset{w\in W}{\text{∑}}x_{p}\delta_{pl}\gamma_{w}\leq g_{l}$∀*l* ∈ *L*(IP 5)*x_p_* = 0 or 1∀*p* ∈ *P_w_*, *w* ∈*W*(IP 6)*y_wl_* = 0 or 1∀*w* ∈ *W*, ∀*l* ∈ *L*.(IP 7)


The objective function is illustrated as (IP) to minimize the mean delay on link *l*. The expression of the form *D_l_*(*g_l_*) is a monotonically increasing and convex function with respect to *g_l_*, where *g_l_* is the aggregate flow on link *l* measured in packets per second [6–9].

#### 3.1.1. Explanation of the Objective Function

Objective function (IP) is actually the summation of average number of packets on each link, *i.e.*, the queue length, which is obtained by the product of link mean delay and aggregate flow on the link. The expression of the form *D_l_*(*g_l_*) is a monotonically increasing and convex function with respect to *g_l_*, where *g_l_* is the aggregate flow on link *l* measured in packets per second [15,21–23].

Through Little's Law (*i.e.*, *N* = λ*T*), the objective function is proportional to the average cross-network packet delay. Thus, for a given traffic input (*i.e.*, $\underset{w\in W}{\text{∑}}\gamma_{w}$), minimizing the average number of packets in the network is equivalent to minimizing the average cross-network packet delay [24,25].

#### 3.1.2. Explanation of the Constraints

QoS constraints:

Constraint (IP 1) confines that the end-to-end delay should be no larger than the maximum allowable end-to-end QoS requirement.

Path constraints:

Constraint (IP 2) confines that all the traffic required by each O-D pair is transmitted over exactly one candidate path.
Constraint (IP 3) confines that once path *p* is selected and link *l* is on the path, *y_wl_* must be equal to 1.

Capacity constraints:

Constraint (IP 4) confines the boundaries of aggregate flow on link *l*.

Flow constraints:

Constraint (IP 5) confines the aggregate flow on link *l* should not exceed the link capacity.

Integer constraints:

Constraint (IP 6) and (IP 7) are the integer constraints of decision variables.

### 3.2. Lagrangian Relaxation

The Lagrangian Relaxation Method was introduced in the 1970s to solve large-scale mathematical programming problems and was followed by a large amount of subsequent research [26,27]. Due to its versatile practicality, the Lagrangian Relaxation Method has become a widely used tool for dealing with optimization problems such as integer programming problems and even non-linear programming problems.

The main idea of the Lagrangian Relaxation method is to pull apart the model by relaxing (*i.e.*, removing) complicated constraints in the primal optimization problem. The next procedure could be modified by the objective function corresponding to the associated Lagrangian multipliers of the relaxed constraints. The primal optimization problem can be transformed into a Lagrangian Relaxation form. The Lagrangian Relaxation problem is separated into several independent sub-problems for each decision variable or other rules by applying the decomposition method. For sub-problems, we can design some heuristics or algorithms to apply and find the optimal value.

Through the adoption of Lagrangian Relaxation, a complicated programming problem can be viewed as a small set of easy-to-solve problems with side constraints. The method simplifies the original problem by decomposing it into several independent sub-problems, each with their own constraints and each of which can be further solved by other well-known algorithms.

For example, if the problem is a minimization problem, the optimal value of the relaxed constraints is always a lower bound on the optimal value of original problem under the relaxed conditions. The lower bound can be improved by adjusting the set of multipliers iteration by iteration to reduce the gap of the solution between the primal problem and the Lagrangian Relaxation. This procedure is also called the Lagrangian Dual problem.

**Figure 2.**
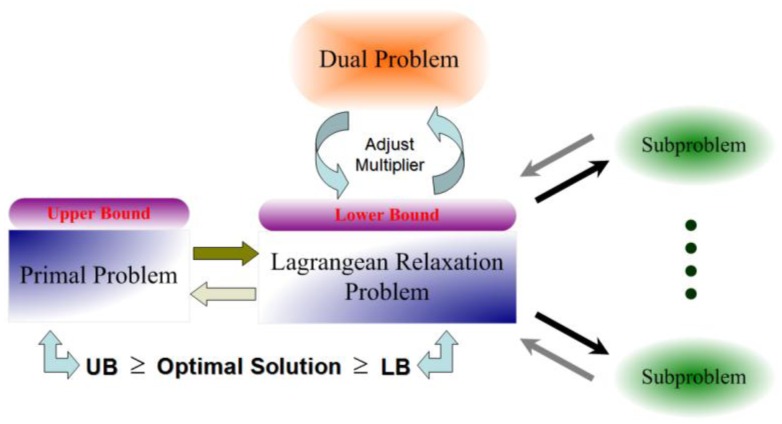
Concept of Lagrangian Relaxation Method.

**Figure 3.**
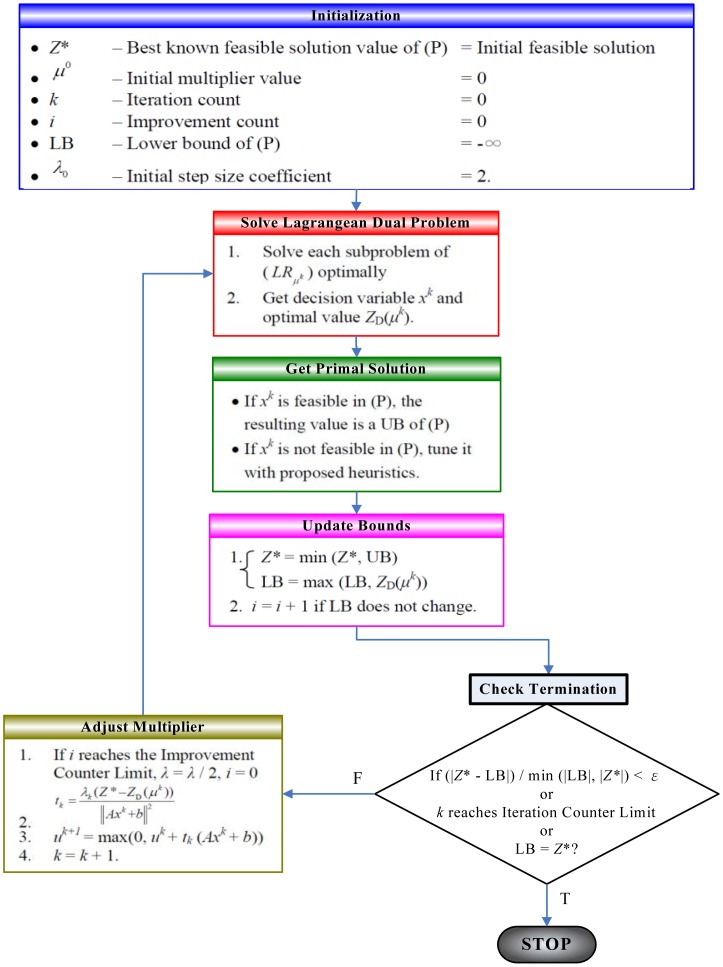
Lagrangian Relaxation Procedures.

Figures 2 and 3 illustrate the underlying idea of the Lagrangian Relaxation Method, respectively. The Lagrangian Relaxation of the primal problem is developed to be a lower bound of the optimal value for the original minimization problem because some constraints of the original problem are relaxed. Therefore, it can be used as the boundary to design heuristic algorithms to get the primal feasible solution. The Subgradient Optimization Method can be utilized to minimize the gap between the primal problem and the Lagrangian Relaxation problem; this can be done through deriving the tightest lower bound by adjusting the multipliers for each iteration and then updating them to improve results.

## 4. Solution Approach

The solution approach to the problem formulation is based on Lagrangian Relaxation. Constraints (IP 1), (IP 3) and (IP 5) are relaxed and multiplied by nonnegative Lagrangian multipliers, $\mu_{w}^{1}$, $\mu_{l}^{2}$, _and_ $\mu_{wl}^{3}$, respectively. They are added to the objective functions as follows.

The constraints are relaxed in such a way that the corresponding Lagrangian multipliers $\mu_{w}^{1}$, $\mu_{l}^{2}$, and $\mu_{wl}^{3}$, can be relaxed to objective function. This approach is not guaranteed to have a feasible solution due to accessing a big value of *γ_w_* into the network which will result in unsatisfactory QoS requirement to the O-D pair *w*.



$\begin{array}{l} Z_{LR}(\mu_{w}^{1},\mu_{l}^{2},\mu_{wl}^{3})=\\ \min\underset{l\in L}{\text{∑}}D_{l}(g_{l})g_{l}\\ +\underset{w\in W}{\text{∑}}\mu_{w}^{1}(\underset{l\in L}{\text{∑}}D_{l}(g_{l})y_{wl}-D_{w})\\ +\underset{l\in L}{\text{∑}}\mu_{l}^{2}(\underset{p\in P_{w}}{\text{∑}}\underset{w\in W}{\text{∑}}x_{p}\delta_{pl}\gamma_{w}-g_{l})\\ +\underset{w\in W}{\text{∑}}\underset{l\in L}{\text{∑}}\mu_{wl}^{3}(\underset{p\in P_{w}}{\text{∑}}x_{p}\delta_{pl}-y_{wl}) \end{array}$(LR)**Subject to:**
$\underset{p\in P_{w}}{\text{∑}}x_{p}=1$∀*w* ∈ *W*(IP 2)
$0\leq g_{l}\leq \frac{C_{l}}{I(l)}$∀*l* ∈ *L*(IP 4)*x_p_* = 0 or 1∀*p* ∈ *P_w_*, *w*∈*W*(IP 6)*y_wl_* = 0 or 1∀*w* ∈ *W*, ∀*l* ∈*L*.(IP 7)


We use the Lagrangian Relaxation Method to relax constraints in the formulation and decompose (LR) into two independent sub-problems. In the first Sub-Problem (SP 1), the nonnegative weight is calculated as ( $\mu_{wl}^{3}+\mu_{l}^{2}\gamma_{w}$) on each link. The actual meaning of the multipliers $\mu_{wl}^{3}$ and $\mu_{l}^{2}$ are the link mean delay and the derivative of queue length, respectively; both can be derived from the solution of the problem (LR). By the problem (LR), the multiplier $\mu_{wl}^{3}$ is related to the relaxation of link selection for each O-D pair. When one unit on the decision variable of link selection is added (*i.e.*, *y_wl_*), it generates one unit of the corresponding mean delay as formulated in QoS constraint, but when the problem (LR) is solved, the decision variable *y_wl_* is either 1 or 0 not only one unit, thus, the multiplier $\mu_{wl}^{3}$ is equivalent to the mean delay on the chosen link.

Besides, the link selection is based on the sum of link mean delay *D_l_*(*g_l_*) of each O-D pair which is weighted by $\mu_{w}^{1}$, that is, how much the end-to-end delay will impact the objective function (IP). Moreover, in the problem (SP 2.1′), the first step for solving it is also related to the multiplier $\mu_{wl}^{3}$ and chooses the links for each O-D pair according to $y_{wl}^{*}(g_{l})=\mu_{w}^{1}D_{l}(g_{l})-\mu_{wl}^{3}=00.$ It means that whenever a link is selected for an O-D pair, the link will produce corresponding link mean delay $\mu_{wl}^{3}$ and affect the end-to-end delay constraint which is weighted by multiplier $\mu_{w}^{1}$ in the problem (LR).

On the other hand, the multiplier $\mu_{l}^{2}$ is related to the relaxation of aggregate flow over each link. The physical meaning of $\mu_{l}^{2}$ would be that when $\mu_{l}^{2}$ can be relaxed on the link flow, it will affect the objective function (IP), which means $\mu_{l}^{2}$ would be the sensitivity of the average number of packets over the link with respect to the aggregate flow.

A brief illustration is shown in Table 4, $\mu_{wl}^{3}$ implies the link mean delay of each link, and $\mu_{l}^{2}$ indicates the derivative of queue length. The term *γ_w_* in the arc weight form represents the weighting factor between $\mu_{wl}^{3}$ and $\mu_{l}^{2}$ Thus, this metric consists of the link mean delay and the derivative of queue length, considers both system perspective and user perspective for our distributed routing protocol, and uses the traffic requirement of each O-D pair as the weighting factor to combine these two parameters.

Finally, The LR problem can be decomposed into independent and solvable optimization sub-problems which are developed in a sufficient way one by one. The physical meanings of multipliers are also developed and mentioned above to help us to determine relationships of the link mean delay and derivative of queue length. We can get these important parameters to derive the routing assignments in “system perspective” and “user perspective”. The detail solving steps are illustrated in the Appendix.

In following section, the computational experiments are constructed and implemented to analyze the quality of the solution approach to verify the routing algorithms correctly. The experiments are designed for analyzing the performance of WVSN, if it can be satisfied with lower average end-to-end delay and delay jitter to transmit video data by different algorithms.

## 5. Computational Experiments and Results

### 5.1. Experiment Environment

**Figure 4.**
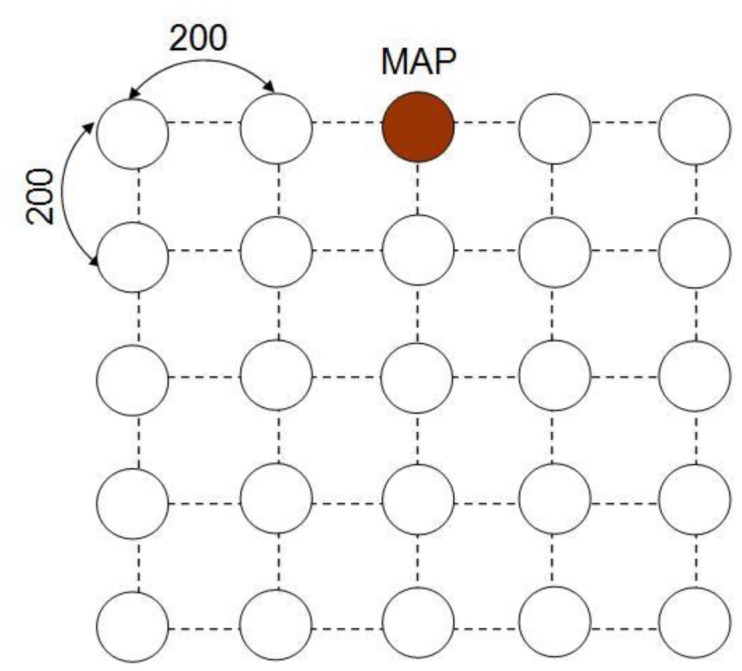
Experimental environment (5 × 5).

Each session follows one path for routing its required traffic emulated as a real time surveillance streaming data and is not allowed to change during the holding time. The session arrivals followed a Poisson process, and the holding time of each session is set to be an exponential distribution with average 10 s. The experiment with average session arrival rates of 0.25, 0.5, 1 and 2, and the corresponding packet arrival rates are 40, 20, 10 and 5. Figure 4 is shown as the experiment environment which QoS requirements are set in 3 × 3, 5 × 5, 7 × 7 and 9 × 9 squares in 3 ms, 4 ms, 5 ms and 6 ms, respectively.

As Table 5 shows, there is average of 100 packets per second in the network of each topology. The size of each transmitting UDP packet is 1,000 bytes at each packet arrival rate. Aside from the control messages of distribution routing protocol, messages such as HELLO messages for sensing the neighbors and TC message for broadcasting topology information are sent at a fixed period of 5 s.

The estimate the parameters of our routing metric (shown in Table 6), we record each packet delay and packet inter-arrival time and then calculate mean delay and aggregate flow with corresponding queue length (*i.e.*, average number of packets) of each link for every second. The number of data fit for power regression is set to be 10, that is, the data used to compute the regression function are the newest 10 records and the interval of each is 1 s. Additionally, the value of *K* in the routing algorithm is set as 5 (*i.e.*, at most the five shortest or fastest paths for each O-D pair) and the threshold *β* for admission control heuristic algorithm is set to be 1.3.

If the received signal strength exceeds the reception threshold, the packet can be successfully received. If received signal strength exceeds the carrier-sensing threshold, the packet transmission can be sensed. However, the packet cannot be decoded unless signal strength surpasses the reception threshold. Both the transmission range and interference range are calculated by the two-ray ground reflection model according to the reception threshold and the carrier-sensing threshold, respectively.

This paper focuses on the effectiveness of our routing metric and related parameters at the on-line stage, and its reduction of system-wide impact between each coming session with QoS provisioning. Thus, we use a single channel in the experimental environment to decouple the effect of the channel assignment algorithm and evaluate the performance of our routing algorithm.

### 5.2. Performance Evaluation

In this Section, we present experimental results to demonstrate the effectiveness of our routing algorithm, NODQC. We also evaluate NODQC with other different session types of algorithms under the same average traffic loading in the network, and then compare the performance in terms of average end-to-end delay, delay jitter and system throughput with QoS satisfaction. Delay jitter is defined as the variance in the following tables and figures.

**Figure 5.**
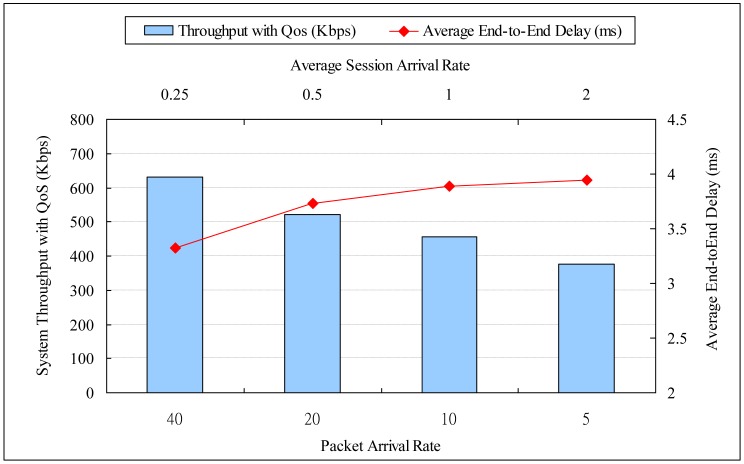
Evaluation with different session arrival rates.

Performance evaluation is defined in Table 7, a 7 × 7 square is simulated as the network topology with 660 s and measure the packets at last 600 s. For comparison with other routing algorithms, we use the session types whose average session arrival rate and packet arrival rate are equal to 0.25 and 40, respectively, and use different network sizes to show the performance of NODQC, OLSR, AODV, and DSDV routing algorithms. The time of our experiment is set at 660 s and the measurement time is the last 600 s. Increasing the number of nodes will also increase the number of broadcast packets and route table updating. From Tables 8 and 9 and Figure 5, the value of average end-to-end delay is shown to be increased and the system throughput with QoS is shown to be decreased with the average session arrival rate. This is because nodes exchange information every 5 s, but the sessions enter the network at a short time interval. Most sessions are consequently routed to sub-optimal paths due to a lack of updated information for the routing metric. Evaluation results indicate that our routing algorithm performs better in the lower average session arrival rate but higher packet arrival rate under the same system loading of average 100 packets per second.

**Figure 6.**
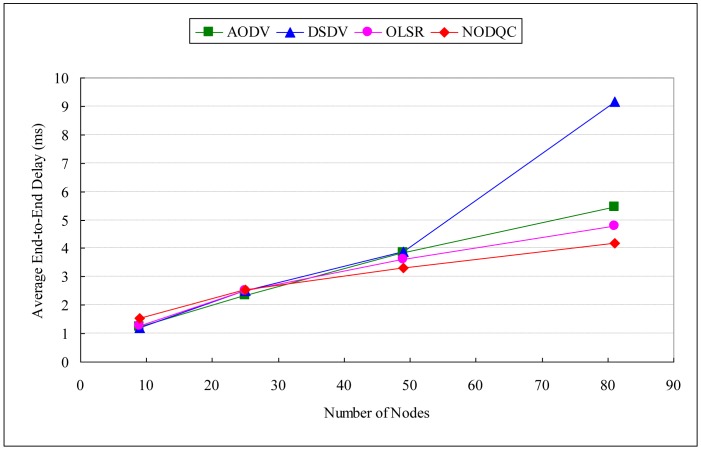
Experiment results of routing algorithms (average end-to-end delay).

**Figure 7.**
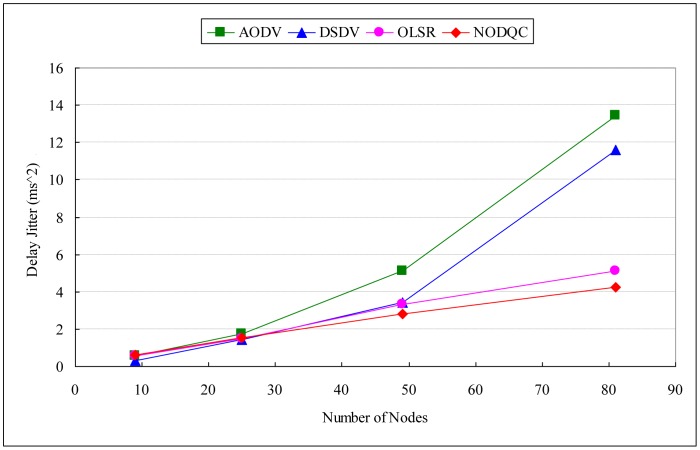
Experiment results of routing algorithms (delay jitter).

**Figure 8.**
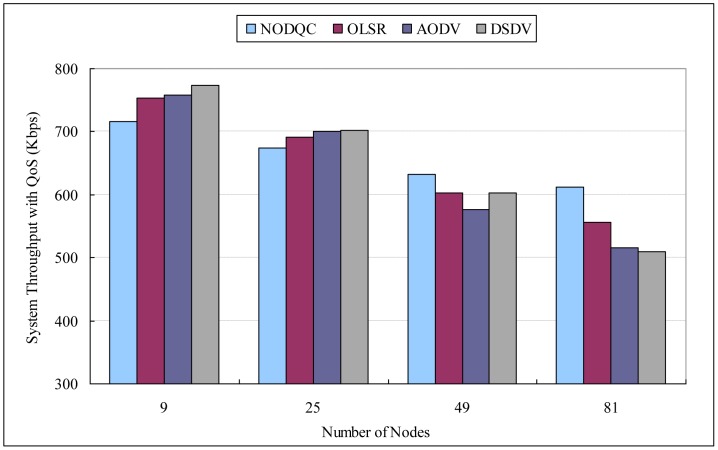
Experiment results of routing algorithms (system throughput with QoS).

If the performance evaluation is taken in the scalability scenario, Average end-to-end delay (AE2ED) shows that it has no effect significant difference in smaller number of nodes obviously, but delay increases as the number of nodes increase. Tables 10, 11 and 12, Figures 6, 7 and 8 show the results of performances between different routing algorithms in each network size. In the small-scale network (e.g., 3 × 3 and 5 × 5 squares), the broadcasting of routing protocol control messages for exchanging information will causes large delays to packet transmission, especially for the destination node. Additionally, the average path length of each session is shorter in smaller networks, in which the superiority of our routing metric is not obvious.

As networks grow in size, the average path of each session lengthens with path selection becoming increasingly important. AE2ED taken in the scalability scenario shows that delay is much less for NODQC as compared to others. As routes break, nodes have to discover new routes which lead to longer E2ED (packets are buffered at the source during route discovery). According to the arc weight on each link is the combination of end-to-end delay from the user perspective and average delay from the system perspective, NODQC can choose other less or non-congested paths for the sessions and balance network-wide the traffic loading. This makes the routing strategy more flexible for making routing decisions for new routing constructions. As far as delay is concerned, NODQC performs better than OLSR, AODV, and DSDV with large numbers of nodes.

Hence for real time traffic, NODQC is preferred over OLSR, AODV, and DSDV. This is significant for the fact that, as the variations of packet delay becomes more predictable, the routing mechanisms can factor in that delay to determine whether a packet is lost or not. Furthermore, the NODQC also takes QoS provisioning into account for the coming sessions, thereby causing system throughput with QoS satisfaction to be greater than others in larger networks. By taking into consideration both the perspective of the system as we as well as users, the NODQC has proven itself to have lower average end-to-end delay and delay jitter and higher system throughput with QoS satisfaction than other routing algorithms in large-scale networks.

By leveraging the Lagrangian Relaxation method, the arc weight on each link can be applied by our routing metrics. It can employ the link-state routing protocol to construct the shortest or shorter paths within an acceptable delay. Within larger networks, routing strategies can be played to improve system performance with lower average end-to-end delay and delay jitter than alternative algorithms (OLSR, AODV, DSDV). NODQC outperforms them in terms of system throughput with QoS satisfaction.

## 6. Future Work

One of the most important parts of this paper is the routing metric and relevant parameters. The Lagrangian Relaxation formulations are applied to the arc weight and to infer the actual meaning of the corresponding multipliers. In addition, Lagrangian multipliers and Karush-Kuhn-Tucker Conditions can be used to succinctly describe the arc weight form. This simplifies the process of inference and has the same consequence with Lagrangian Relaxation.

Through the solution of the Lagrangian Relaxation formulation, various QoS requirements can be implemented for different purposes or services. The end-to-end delay is presented as a function in our formulation to be the QoS consideration. Distinct QoS metrics (e.g., delay jitter or packet loss rate) can be applied to the Lagrangian Relaxation formulation, and the corresponding multiplier of the routing metric, link mean delay will be replaced by the newly defined QoS metric.

## 7. Conclusions

In wireless visual sensor networks for real time video surveillance, sensor nodes need to be forwarded the packets to the sinks within an acceptable delay under limited resource constraints, including embedded vision processing, data communication, battery energy issues. It is a challenging issue since it needs to meet the end-to-end delay for each application and at the same time optimize the system resource utilization by minimizing the average system delay so that more real-time applications could be granted in the future. By leveraging the Lagrangian Relaxation method, the arc weight on each link is the combination of end-to-end delay from the user perspective and average delay from the system perspective. Thereafter, our routing metric employs the link-state routing protocol to construct shortest paths within an acceptable delay. Within larger networks, the superiority of the Near-Optimal Distributed QoS Constrained (NODQC) routing algorithm is more visible as path selection plays a more important role. Experimental results show that, and especially so in large-scale networks, the NODQC not only has lower average end-to-end delay and delay jitter than alternative algorithms (OLSR, AODV, DSDV) but also outperforms them in terms of system throughput with QoS satisfaction. In WVSNs for video surveillance, video coding/compression that has low complexity, produces a low output bandwidth, tolerates loss, and consumes as little power as possible is required. Our routing strategies can be applied well in a large scale network with more efficiency and effectiveness.

## Figures and Tables

**Table 1.** Routing protocol comparison.

**Routing Protocol**	**NODQC**	**AODV**	**OLSR**	**DSDV**
Type	Reactive/On-demand	Reactive/On-demand	Proactive/Table driven	Proactive/Table driven
	Optimization with construct shortest paths within an acceptable delay.	Quick adaptation under dynamic link conditions	Optimization: MultiPoint Relays (MPRs)	
			Forward broadcast messages during the flooding process	Adds two things to distance-vector routing
Short Description	Lower average end-to-end delay and delay jitter	Lower transmission latency	Only partial link state is distributed	Sequence number; avoid loops
	Higher system throughput with QoS satisfaction in large networks	Consume less network bandwidth (less broadcast)	Optimal routes (in terms of number of hops)	Damping; hold advertisements for changes of short duration
	Scalable to large networks	Loop-free property	Suitable for large and dense networks	
Distributed	Yes	Yes	Yes	Yes
Unidirectional Link Support	Yes	No	Yes	No
Periodic Broadcast	Yes	Yes	Yes	Yes
QoS Support	Yes	No	Yes	No
Advantages	Arc weight of link can be determined and prioritized			
	Faster decision making	Lower connection setup time than DSR		Simplicity
	Suitable for large and dense networks	Sequence numbers used for route freshness and loop prevention		Lower route request latency, but higher overhead
	Construct shortest paths within an acceptable delay.		Suitable for large and dense networks	Incremental dumps and settling time used to reduce control overhead
	Lower average end-to-end delay and delay jitter	Maintains only active routes	Independent from other protocols	
	Higher system throughput with QoS satisfaction in large networks	Uses sequence numbers to determine route age to prevent usage of stale routes		Perform best in network with low to moderate mobility, few nodes and many data sessions
	Low complexity with more efficiency and effectiveness			
Disadvantages	Constrains must be relaxed	Link break detection adds overhead	Lack of security	Count-to-infinity problem
	Proprietary design Applications oriented	Still have possible latency before data transmission can begin	No support for multicast	Not efficient for large Ad hoc networks
			Overhead: maintaining routes to all nodes is often unnecessary	Routing information is broadcasted periodically and incrementally

**Table 2.** Given parameters.

**Notation**	**Description**
*W*	The set of Origin-Destination (O-D) pairs in the WSNs, where *w* ∈ *W*.
*P_w_*	The set of directed paths from the origin to the destination of O-D pair *w*, where *p* ∈ *P_w_*.
*L*	The set of communication links in the WSNs, where *l* ∈ *L*.
*I*(*l*)	The number of interference links of link *l*.
*δ_nl_*	The indicator function which is 1 if link *l* is on path *p* and 0 otherwise.
*C_l_*	(*packets*/*s*) The link capacity of link *l*.
*γ_w_*	(*packets*/*s*) The given traffic input of O-D pair *w*.
*D_l_*(*g_l_*)	The mean delay on link *l*, which is a monotonically increasing and convex function of aggregate flow *g_l_*.
*D_w_*	The maximum allowable end-to-end QoS for O-D pair *w*.

**Table 3.** Decision variables.

**Notation**	**Description**
*x_p_*	1 if path *p* is used to transmit the packets for O-D pair *w* and 0 otherwise.
*y_wl_*	1 if link *l* is on the path *p* adopted by O-D pair *w* and 0 otherwise.
*g_l_*	(*packets*/*s*) The estimate of the aggregate flow on link *l*.

**Table 4.** Arc weight of each link.

$\begin{array}{c} \mathrm{arc weight}=(\mu_{wl}^{3}+\mu_{l}^{2}\gamma_{w})\\ =\text{link mean delay}+\text{derivative of queue length}\times \text{required traffic}\\ =D_{l}(g_{l})+\gamma_{w}\times \frac{\partial \left(D_{l}(g_{l})g_{l}\right)}{\partial g_{l}}\\ =D_{l}(g_{l})+\gamma_{w}\times \left(D_{l}(g_{l}+g_{l}\times \frac{\partial D_{l}(g_{l})}{\partial g_{l}}\right) \end{array}$

**Table 5.** Experimental session types.

**Parameters**	**Value**			
Average Holding Time	10			
Average Session Arrival Rate	0.25	0.5	1	2
Average Number of Active Sessions	2.5	5	10	20
Packet Arrival Rate	40	20	10	5
Average Traffic Input	100 (packets/s)			

**Table 6.** Parameters.

**Parameters**	**Value**
Transmit and Receive Antenna Gain	1.0
Transmit and Receive Antenna Height	1.5 (m)
Reception Threshold	3.625 e^−;10^
Carrier Sensing Threshold	1.559 e^−;11^
Transmission Range	250 (m)
Interference Range	550 (m)
Distance between Each Node	200 (m)
UDP Packet Size	1,000 (bytes)
Sending Interval of HELLO Message	2 (s)
Sending Interval of TC Message	5 (s)
Recording Interval of Fitting Data	1 (s)
Number of Fitting Data	10
K	5
*β*	1.3

**Table 7.** Two-Ray Ground Reflection Model.

**Transmission Range (or Interference Range)**
$\sqrt[{4}]{\frac{\text{TransmitPower}\times \text{TransmitAntennaGain}\times \text{ReceiveAntennaGain}\times {\left(\text{TransmitAntennaHeight}\right)}^{2}\times {\left(\text{ReceiveAntennaHeight}\right)}^{2}}{\text{Threshold}}}$

**Table 8.** Evaluation with different session rates (average end-to-end delay).

**Average End-to-End Delay (ms)**				
Average Session Rate	0.25	0.5	1	2
Packet Arrival Rate	40	20	10	5
Average End-to-End Delay	3.325215	3.730487	3.88681	3.942485

**Table 9.** Evaluation with different session rates (system throughput with QoS).

**System Throughput with QoS (Kbps)**				
Average Session Rate	0.25	0.5	1	2
Packet Arrival Rate	40	20	10	5
System Throughput with QoS	632.062518	521.353393	456.291533	376.553628

**Table 10.** Experiment results of routing algorithms (average end-to-end delay).

**Average End-to-End Delay (ms)**				
Routing Algorithms	3 × 3	5 × 5	7 × 7	9 × 9
NODQC	1.54599	2.550296	3.325215	4.176691
OLSR	1.28128	2.522533	3.620345	4.792818
AODV	1.248075	2.339906	3.848888	5.456055
DSDV	1.220291	2.495117	3.873495	9.171553

**Table 11.** Experiment results of routing algorithms (delay jitter).

**Delay Jitter (ms^2^)**				
Routing Algorithms	3 × 3	5 × 5	7 × 7	9 × 9
NODQC	0.623741	1.529524	2.789917	4.239667
OLSR	0.536928	1.463894	3.332403	5.098757
AODV	0.556566	1.720252	5.129518	13.445979
DSDV	0.330232	1.418664	3.424205	11.586675

**Table 12.** Experiment results of routing algorithms (system throughput with QoS).

**System Throughput with QoS (Kbps)**				
Routing Algorithms	3 × 3	5 × 5	7 × 7	9 × 9
NODQC	716.849085	673.698465	632.062518	612.322159
OLSR	753.008325	691.484223	603.130035	556.022380
AODV	757.683145	700.203701	575.904857	516.320711
DSDV	773.355103	701.779457	602.953338	509.221205

**Figure A1.**
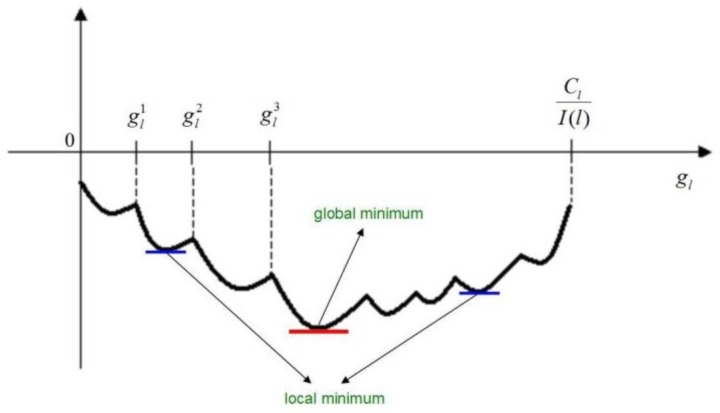
Typical graph of problem (SP 2.1′).

**Figure A2.**
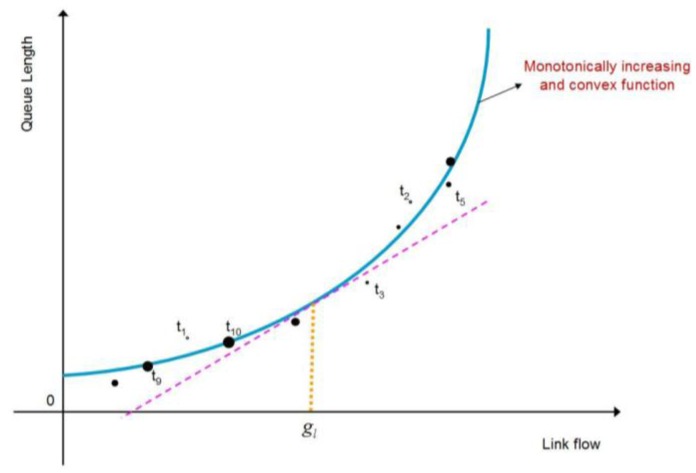
Estimation of the derivative of queue length.

**Figure A3.**
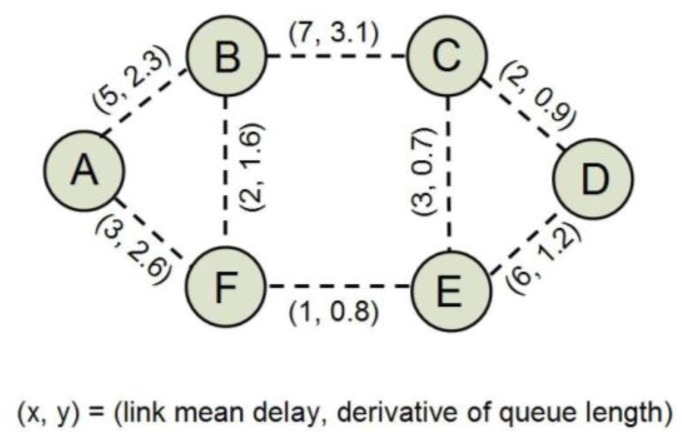
Simple networks with weight information of each link.

**Figure A4.**
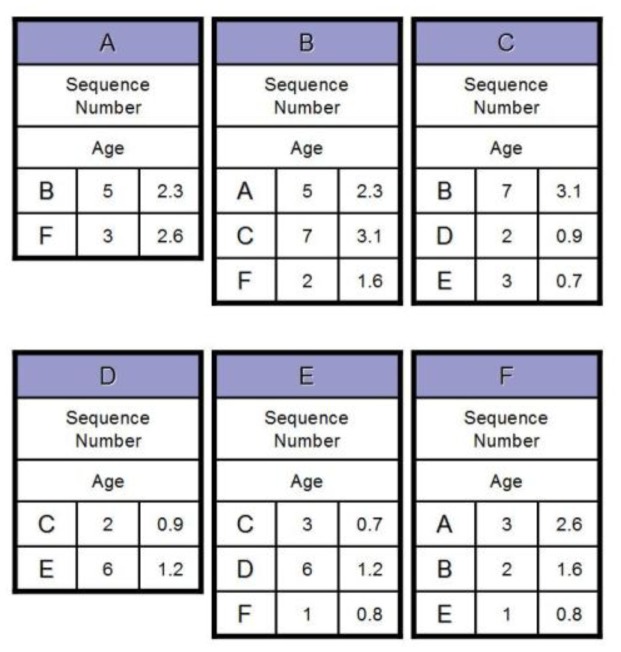
Link state packets for all nodes in simple networks.

## Appendix

### Problem Solving Steps

The solution approach to the problem formulation is based on Lagrangian Relaxation. Constraints (IP 1), (IP 3) and (IP 5) are relaxed and multiplied by nonnegative Lagrangian multipliers, respectively. They are added to the objective functions as follows:

$\begin{array}{l} Z_{LR}(\mu_{w}^{1},\mu_{l}^{2},\mu_{wl}^{3})=\\ \min\underset{l\in L}{\text{∑}}D_{l}(g_{l})g_{l}\\ +\underset{w\in W}{\text{∑}}\mu_{w}^{1}(\underset{l\in L}{\text{∑}}D_{l}(g_{l})y_{wl}-D_{w})\\ +\underset{l\in L}{\text{∑}}\mu_{l}^{2}(\underset{p\in P_{w}}{\text{∑}}\underset{w\in W}{\text{∑}}x_{p}\delta_{pl}\gamma_{w}-g_{l})\\ +\underset{w\in W}{\text{∑}}\underset{l\in L}{\text{∑}}\mu_{wl}^{3}(\underset{p\in P_{w}}{\text{∑}}x_{p}\delta_{pl}-y_{wl}) \end{array}$		(LR)
**Subject to:**		
$\underset{p\in P_{w}}{\text{∑}}x_{p}=1$	∀*w* ∈ *W*	(IP 2)
$0\leq g_{l}\leq \frac{C_{l}}{I(l)}$	∀*l* ∈ *L*	(IP 4)
*x_p_* = 0 or 1	∀*p* ∈ *P_w_,w* ∈ *W*	(IP 6)
*y_wl_* = 0 or 1	∀*w* ∈ *W*, ∀*l* ∈ *L*.	(IP 7)

The constraints are relaxed in such a way that the corresponding Lagrangian multipliers $\mu_{w}^{1}$, $\mu_{l}^{2}$, and $\mu_{wl}^{3}$ are non-negative. This approach is not guaranteed to have a feasible solution due to accessing a big value of *γ_w_* into the network which will result in unsatisfactory QoS requirement to the O-D pair *w*. However, LR can be decomposed into the following two independent and solvable optimization sub-problems in a sufficient way.

Step 1: Sub-problem 1 (related to *x_p_*)

**Objective Function:**		
$\begin{array}{l} Z_{\text{sub}1}(\mu_{l}^{2},\mu_{wl}^{3})\\ =\min\underset{w\in W}{\text{∑}}\underset{p\in P_{w}}{\text{∑}}\underset{l\in L}{\text{∑}}(\mu_{wl}^{3}+\mu_{l}^{2}\gamma_{w})x_{p}\delta_{pl} \end{array}$		(SP 1)
**Subject to:**		
$\underset{p\in P_{w}}{\text{∑}}x_{p}=1$	∀*w* ∈ *W*	(IP 2)
*x_p_* = 0 or 1	∀*p* ∈ *P_w_, w* ∈ *W*	(IP 6)
*y_wl_* = 0 or 1	∀*w* ∈ *W*, ∀*l* ∈ *L*.	(IP 7)

The problem can be further decomposed into |*W*| independent shortest path problems. Each shortest path problem can be easily solved by Dijkstra's Algorithm with nonnegative arc weights. ( $\mu_{wl}^{3}+\mu_{l}^{2}\gamma_{w}$) is the exact arc weight determined from our distributed routing protocol.

Step 2: Sub-problem 2 (related to *y_wl_* and *g_l_*)

**Objective Function:**		
$\begin{array}{l} Z_{\text{sub}2}(\mu_{w}^{1},\mu_{l}^{2},\mu_{wl}^{3})\\ =\min[\underset{l\in L}{\text{∑}}(D_{l}(g_{l})g_{l}+\underset{w\in W}{\text{∑}}\mu_{w}^{1}y_{wl}D_{l}(g_{l})-\mu_{l}^{2}g_{l}-\underset{w\in W}{\text{∑}}\mu_{wl}^{3}y_{wl})-D_{w}\underset{w\in W}{\text{∑}}\mu_{w}^{1}] \end{array}$		(SP 2)
**Subject to:**		
$0\leq g_{l}\leq \frac{C_{l}}{I(l)}$	∀*l* ∈ *L*	(IP 4)
*y_wl_* = 0 or 1	∀*w* ∈ *W*, ∀*l* ∈ *L*.	(IP 7)

In objective function (SP 2), the last term $-D_{w}\underset{w\in W}{\text{∑}}\mu_{w}^{1}$ is not changed to determine the optimal solution. It can be disregarded first and then added back to the objective value. Therefore, (SP 2) can be reformulated and further decomposed into |*L*| independent sub-problems. The objective function (SP 2) can be determined to function (SP 2.1) for each link *l*.

$\min[D_{l}(g_{l})g_{l}+\underset{w\in W}{\Sigma }\mu_{w}^{1}y_{wl}D_{l}(g_{l})-\mu_{l}^{2}g_{l}-\underset{w\in W}{\Sigma }\mu_{wl}^{3}y_{wl}]$		(SP 2.1)
**Subject to:**		
$0\leq g_{l}\leq \frac{C_{l}}{I(l)}$	∀*l* ∈ *L*	(IP 4)
*y_wl_* = 0 or 1	∀*w* ∈ *W*, ∀*l* ∈ *L*.	(IP 7)

In problem (SP 2.1), the first term *D_l_*(*g_l_*)*g_l_* is a monotonically increasing and nonnegative function. If the minimum value of the other terms in (SP 2.1) can be determined, the first term *D_l_*(*g_l_*)*g_l_* can be eliminated in the first step. (SP 2.1) can be expressed as (SP 2.1′) for each link *l*,

$\min[\underset{w\in W}{\Sigma }\mu_{w}^{1}y_{wl}D_{l}(g_{l})-\mu_{l}^{2}g_{l}-\underset{w\in W}{\Sigma }\mu_{wl}^{3}y_{wl}]$		(SP 2.1′)
**Subject to:**		
$0\leq g_{l}\leq \frac{C_{l}}{I(l)}$	∀*l* ∈ *L*	(IP 4)
*y_wl_* = 0 or 1	∀*w* ∈ *W*, ∀*l* ∈ *L*.	(IP 7)

**Table A1.** Algorithm for solving problem (SP 2.1′).

Step 1: Solve $y_{wl}^{*}(g_{l})=\mu_{w}^{1}D_{l}(g_{l})-\mu_{wl}^{3}=0$for each O-D pair *w*, and define the results as a set of break points of *g_l_*. Step 2: Sort these break points and denote as $g_{l}^{1}$, $g_{l}^{2}$, $g_{l}^{n}$ There are at most |*W*| break points. Step 3: At each interval $g_{l}^{i}\leq g_{l}\leq g_{l}^{i+1}$, $y_{wl}^{*}(g_{l})$ is 1 if $\mu_{w}^{1}D_{l}(g_{l})-\mu_{wl}^{3}\leq 0$ and is 0 otherwise. Step 4: Denote $\underset{w\in W}{\text{∑}}\mu_{w}^{1}y_{wl}^{*}(g_{l}^{i})$as *a_l_* and $\underset{w\in W}{\text{∑}}\mu_{wl}^{3}y_{wl}^{*}(g_{l}^{i})$as *b_l_*, within the interval $g_{l}^{i}\leq g_{l}\leq g_{l}^{i+1}$, the problem (SP 2.1′) can be expressed as: $a_{l}D_{l}(g_{l})-\mu_{l}^{2}g_{l}-b_{l}.$Thus, the local minimum is either the boundary point, $g_{l}^{1}$or $g_{l}^{i+1}$, or at point $g_{l}^{*}$, where $g_{l}^{*}$is the solution to $a_{l}\frac{\partial D_{l}(g_{l})}{\partial g_{l}}-\mu_{l}^{2}=0.$. Step 5: By examine the |*W*| +; 1 intervals, the global minimum point can be found by comparing these local minimum points.

Finally, this problem can be solved by the algorithm developed by Cheng and Lin in [28]. The solution steps and graphics of the algorithm are briefly described in Table A1 and Figure A1.

Step 3: The Dual Problem and the Subgradient Method

According to the Weak Lagrangian Duality Theorem [29], for the $\mu_{w}^{1}$, $\mu_{l}^{2}$, $\mu_{wl}^{3}$ ⩾ 0, the objective value of the Lagrangian Relaxation problem *Z_LR_* ( $\mu_{w}^{1}$, $\mu_{l}^{2}$, $\mu_{wl}^{3}$) is a lower bound of the primal problem *Z_IP_*. Based in problem (LR), the following dual problem *Z_D_* is constructed to calculate the tightest lower bound.

**Dual Problem (D):**	
*Z_D_* = max *Z_D_*( $\mu_{w}^{1}$, $\mu_{l}^{2}$, $\mu_{wl}^{3}$)	(D)
**Subject to:**	
$\mu_{w}^{1}$, $\mu_{l}^{2}$, $\mu_{wl}^{3}$⩾ 0.	

We use the Subgradient Method is used to solve that [26,27] to solve the dual problem (D). First, let the vector *S* be a subgradient of $Z_{D}(\mu_{w}^{1},\mu_{l}^{2},\mu_{wl}^{3})$. In iteration *k* of the Subgradient Optimization Procedure, the multiplier vector *π^k^* = ( $\pi^{k}=(\mu_{w}^{1},\mu_{l}^{2},\mu_{wl}^{3})$ is updated by *π^k^*^+;1^ = *π^k^* +; *t^k^S^k^*. The step size *t^k^* is determined by $t^{k}=\lambda \frac{\left(Z_{IP}^{h}-Z_{D}(\pi^{k})\right)}{{\left\|S^{k}\right\|}^{2}}$. $Z_{IP}^{h}$ is the primal objective function value (an upper bound on *Z_IP_*) and *λ* is constant where 0 ≤ λ ≤ 2.

Step 4: Getting Primal Feasible Solutions

A primal feasible solution to (IP) by definition must also be a solution to (LR) and satisfy all constraints. Otherwise, it must be modified to be feasible to (IP) by a getting primal feasible solutions heuristic. According to the computational experiments in [21], the heuristic can be better solutions that consider the end-to-end delay constraints. That is, if the end-to-end delay constraints are not satisfied, more arc weights along those paths violate the end-to-end delay constraints in which case routing assignments must then be recalculated. Note that, this approach requires a few minutes to obtain the feasible solution or even the near-optimal solution. However, this paper focuses on the dynamic network environment and the derivation of the arc weight. Therefore, this arc weight will be our routing metric used by the distributed routing protocol for dynamic routing.

Step 5: Routing Metric

The cost of passing through the network can be used as a metric. By virtue of the routing algorithm, each router chooses the path with the smallest (shortest) sum of the metrics. Different routing protocols define the metric distinctly. The metric can be distance, number of hops, delay, and so on. Here, the metric is defined as a combination of the link mean delay and the derivative of queue length for each link.

Step 6: Estimation of Routing Metric Parameters

#### Perturbation Analysis

In [25], Towsley *et al.* proposed “Perturbation Analysis” (PA), a useful technique for estimating the so-called “marginal delay” defined in [24]. More specifically, this method is used to estimate the derivative of queue length. As previously mentioned, the derivative of queue length is composed of link mean delay, aggregate flow, and the partial derivative of link mean delay. The PA approach can also estimate the value of link mean delay, a step that precedes the calculation of the derivative of queue length. One can use the PA technique to determine the link mean delay and the derivative of queue length, both major components of the distributed routing protocol.

#### Time Stamping for Packets

Other approaches can also estimate critical information of each link in a conceptually straightforward way. During packet reception, packet delay time is defined as the difference between sending time and receiving time. For each adjacent link mean delay, it the sum of each packet delay divided by the number of received packets corresponding to each neighbor node.

#### Approximation Function

For the derivative of queue length, queue length and the corresponding aggregate flow over the link can be recorded at each time interval. Figure A2 is illustrated a monotonically increasing and convex function such as the power regression function (*i.e.*, *y* = *ax^b^*, where *a* > 0 and *b* > 1) can be used to approximate the recording results, and the newest record has the most impact to this approximation. Therefore, the derivative of queue length can be obtained by calculating the derivative of the function with respect to the corresponding aggregate flow on the link.

Step 7: Distributed Routing Algorithm

The routing protocol is proposed and based on the well-known link-state routing protocol [16]. Link-state routing protocol assigns a cost (*i.e.*, metric) to each route. The link mean delay is provided to be a metric by the present routing protocol. The derivative of queue length is added to the information exchanged by each node for realizing the traffic flow status and estimation.

The underlying idea behind this type of routing is that each node shares the state of its neighborhood to every other node in the network. By exchanging local information with all other nodes, each node will have all link states in its own database [16]. In other words, every node has access to the entire network topology including the weight information of each link. Link state packets for all nodes in Figure A3 are shown in Figure A4.

The underlying idea behind this routing protocol is that only a few changes in present link-state routing protocol apply to our routing algorithm. Each node must do the following steps in Table A2.

Whenever a route from source to any destination in the network is required, the source node computes the arc weight for each link by the required traffic and pertinent information of each link in the link state database.

**Table A2.** Distributed routing protocol for each node.

Step 1: Discover its neighbors and learn their network address. Step 2: Measure the link mean delay and the derivative of queue length to each of its neighbors. Step 3: Build a packet with the above information at regular intervals. Step 4: Flood this packet to all other node in the network. Step 5: Compute the shortest path based on the arc weight form to every other node. Step 6: Flood the path results to all other nodes included in the path.

Step 8: Admission Control Heuristic Algorithm

Through the link-state routing protocol, the Dijkstra Algorithm can be applied to each node to calculate the routing table and can also compute the shortest path between any two nodes in the network. The algorithm denotes two states for the nodes, tentative and permanent. It is briefly described the steps of Dijkstra algorithm in Table A3.

**Table A3.** Dijkstra routing algorithm [16].

Step 1: Start with the local node (*i.e.*, the root of the tree). Step 2: Assign a cost of 0 to this node and make it the first permanent node. Step 3: Examine each neighboring node of the last permanent node. Step 4: Assign a cumulative cost to each node and make it tentative. Step 5: Among the list of tentative nodes Step 5.1: Find the node with the smallest cumulative cost and make it permanent. Step 5.2: If a node can be reached from more than one direction Step 5.2.1: Select the direction with the shortest cumulative cost. Step 6: Repeat Step 3 to Step 5 until every node becomes permanent.

To keep our routing as flexible as possible, the Dijkstra Algorithm only computes shortest path for each node pair. Nevertheless, in this paper, it is also important to construct the second shortest path, the third shortest path, and so on; that is so called “K shortest paths.” A significant amount of research has centered on the K shortest paths problem as mentioned in [14,30], and Katoh *et al.* in [31] proposes the adoption of the K shortest paths (KSP) algorithm. The algorithm is suitable for an undirected graph with nonnegative arc weight and can compute K simple paths (*i.e.*, without cycles between the paths) in *O*(*Kc*(*n*,*m*)) time. Where *c*(*n*,*m*) is the time to compute shortest paths from one node to all the other nodes?

The experiment environment is constructed in four grid topologies of 3 × 3, 5 × 5, 7 × 7 and 9 × 9 squares, with one node placed in each intersection point as shown in Figure 5. Each sensor node has a radio transmission range of 250 m and a radio interference range of 550 m. The only sink is located on the center of the first row in each square with sessions randomly generated by the other nodes in the topology. The ns-2 simulator evaluates the performance of the NODQC routing algorithm with different types of routing algorithms.

This means that, if the Dijkstra Algorithm is the shortest path algorithm, the complexity of the K shortest paths algorithm will be *O*(*Kn^2^*). Furthermore, metrics used for calculating the candidate shortest paths not only include arc weight, but also the predictive link mean delay. In other words, there are two types of the “K shortest paths”. If not all K shortest paths calculated by the original arc weight can satisfy the end-to-end delay requirement, the predictive link mean delay will be the metric for each link and calculate the corresponding K shortest paths, *i.e.*, the K fastest paths, to satisfy the maximum allowable QoS requirements as possibly as we can.

By determining both the K shortest paths and the K fastest paths, our admission control heuristic algorithm is therefore constructed to give due consideration to both the “system perspective” and “user perspective”. Unfortunately, the K shortest paths may still not satisfy the basic assumption, the QoS provisioning. Thus, other K fastest paths are provided to meet the QoS requirement, and the original arc weight corresponding to each of which cannot exceed a threshold *β* which confines the effect to the whole system. The steps are shown in detail in Table A4.

**Table A4.** Admission control heuristic algorithm.

Step 1: Calculate the fastest path by the predictive link mean delay as the arc weight. Step 1.1: If the fastest path can't satisfy the QoS requirement. Step 1.1.1: Reject this traffic. Step 2: Calculate K shortest paths by the arc weight we proposed, and denote *θ* as the cost of first shortest path. Step 3: Compare the QoS requirement to the predictive end-to-end delay of each K shortest paths. Step 3.1: If one of the K shortest path can satisfy the QoS requirement Step 3.1.1: Choose the shortest one for routing this traffic. Step 4: Calculate K fastest paths by the predictive link mean delay as the weight of each link, and set *α_k_* to be the original length corresponding to the K-th fastest path. Step 4.1: If the K-th fastest path can satisfy the QoS requirement and $\frac{\alpha_{K}}{\theta }\leq \beta$ Step 4.1.1: Choose the fastest one (*i.e.*, smallest K), and route this traffic. Step 5: Reject this traffic.

The power regression function can predict the link mean delay of each link. Through admission control heuristic algorithm, any required traffic unable to satisfy the QoS requirement will be rejected with the predictive end-to-end delay. This mechanism therefore reduces system-wide impact can be reduced and other flows in advance and ultimately achieve superior performance.
